# Draft genome sequence of *Serratia marcescens* NGJE-81: a drain sediment isolate from Bangladesh

**DOI:** 10.1128/mra.01008-25

**Published:** 2026-01-20

**Authors:** Fahad Khan, Abdus Sadique, Md. Mashiur Rahaman, Nayma Haque Tonny, Jahidul Alam, Muhsina Tabassum, Tasluma Tamanna, Solaiman Miah, Fariza Shams, Ayesha Sharmin, Ali Azam Talukder, Baki Billah, Md. Mahbubur Rahman, Sayad Md. Didarul Alam, Md. Mainul Hossain, Maqsud Hossain

**Affiliations:** 1 NSU Genome Research Institute (NGRI), North South University 54495, Dhaka, Bangladesh; 2 Department of Biochemistry & Microbiology, School of Health and Life Sciences, North South University 54495, Dhaka, Bangladesh; 3 Department of Chemistry, Bangladesh University of Engineering & Technology, Dhaka, Bangladesh; 4 Department of Microbiology, Jahangirnagar University, Dhaka, Bangladesh; 5 Department of Pharmaceutical Science, School of Health and Life Sciences, North South University 54495, Dhaka, Bangladesh; California State University San Marcos, San Marcos, California, USA

**Keywords:** *Serratia marcescens*, draft genome sequencing, environmental isolate, antimicrobial resistance genes, opportunistic pathogen

## Abstract

Here, we report the draft genome sequence of *Serratia marcescens* NGJE-81 isolated from drain sediment. The genome consists of approximately 4.95 Mb with a G + C content of 59.79%. This genome sequence will provide insights into the genetic basis of this organism’s versatile metabolism and potential biotechnological applications.

## ANNOUNCEMENT

*Serratia marcescens* is a distinctive opportunistic pathogen characterized by its ability to produce a red pigment called prodigiosin and its remarkable adaptability to diverse environments, ranging from soil and water to healthcare settings and medical devices (1, 2). The organism has gained significant clinical importance due to its intrinsic resistance to multiple antibiotics and its emergence as a causative agent of various healthcare-associated infections (3, 4). The strain NGJE-81 was isolated from soil samples collected from drain sediment in the canteen in Jahangirnagar University, Dhaka, Bangladesh (23.8793, 90.2707) on 24 February 2019. This strain was selected for sequencing due to its distinctive red-pigmented phenotype, environmental origin from a drain sediment, and preliminary indications of antimicrobial resistance, alongside the scarcity of genomic data for environmental *S. marcescens* isolates from Bangladesh. The sample was first inoculated in LB broth media and incubated at 37°C overnight for enrichment. It was then streaked on MacConkey Agar media and incubated overnight (~18 h) at 37°C. A single visible contamination-free colony was selected for further analysis including DNA extraction and stocking in 30% Glycerol + LB Broth for long-term preservation.

A single colony grown overnight in LB broth was used for genomic DNA extraction using the Promega Wizard Genomic DNA Purification Kit (Wisconsin, USA), following the manufacturer’s protocol. The genome was then sequenced using the Illumina MiSeq platform with paired-end reads (2 × 150 bp) approach using the MiSeq Reagent Kit V3 (Illumina Inc. USA) at the NSU Genome Research Institute. Library preparation was done using the Nextera XT Library preparation kit. A total of 685,085 paired-end reads were generated totaling 100.7 Mbp. Quality control of the raw reads was performed using FastQC v0.12.1 (5), and trimming was done using Trim Galore v0.6.7 (6).

The genome was assembled using SPAdes v3.15.0 (7) (cutoff = 30) with the trimmed sequences resulting in 36 contigs with an N50 value of 412,400 bp. The draft genome sequence consists of 4,951,510 bp with a G + C content of 59.79%. Analysis through KmerFinder-3.2 server (7) confirmed the sample to be *Serratia marcescens*. The genome assembly demonstrated excellent completeness with BUSCO v5.8.0 (8) analysis, revealing 99.8% (439) of the 440 searched ortholog groups (data set: enterobacterales_odb10) being present and complete as single-copy genes, with only one BUSCO missing, indicating a high-quality genome assembly. Genome annotation was performed using both Prokka v1.14.6 (9) and NCBI’s Prokaryotic Genome Annotation Pipeline (PGAP) v6.9 (10). PGAP predicted the genome to contain 4,729 genes, of which 4,600 genes are protein coding, including genes coding for 5 rRNAs and 79 tRNAs. While Prokka annotation predicted 4,550 genes, 4 rRNAs, 84 tRNA genes, and 1 tmRNA. Antimicrobial resistance gene analysis using ResFinder v4.7.2 (11) identified three resistance genes in the genome, aac(6′)-Ic, blaSST-1, and tet(41). The aac(6′)-Ic gene confers aminoglycoside resistance, blaSST-1 encodes a β-lactamase for β-lactam resistance, and tet(41) mediates tetracycline resistance via an efflux pump. Further analysis using PathogenFinder2 (12) predicted the bacteria to be a potential human pathogen with a mean score of 0.9678. All tools used in this study were run with default parameters unless otherwise mentioned. These findings can enhance our understanding of *S. marcescens* NGJE-81 and contribute to future research on its role in environmental and clinical settings.

## Data Availability

The genome sequence is available at NCBI GenBank under the accession JBLQTD000000000, with raw reads in the NCBI SRA database, SRR32147338. The study is registered under the BioProject PRJNA1216405. The Prokka, ResFinder, and pathogen finder output files have been published on Figshare and are available at (DOI: https://doi.org/10.6084/m9.figshare.30750974).
